# Mapping the route from naive pluripotency to lineage specification

**DOI:** 10.1098/rstb.2013.0540

**Published:** 2014-12-05

**Authors:** Tüzer Kalkan, Austin Smith

**Affiliations:** 1 Wellcome Trust–Medical Research Council Cambridge Stem Cell Institute, University of Cambridge, Tennis Court Road, Cambridge CB2 1QR, UK; 2 Department of Biochemistry, University of Cambridge, Tennis Court Road, Cambridge CB2 1QR, UK

**Keywords:** pluripotency, embryonic stem cells, lineage specification, signalling, epiblast, self-renewal

## Abstract

In the mouse blastocyst, epiblast cells are newly formed shortly before implantation. They possess a unique developmental plasticity, termed naive pluripotency. For development to proceed, this naive state must be subsumed by multi-lineage differentiation within 72 h following implantation. *In vitro* differentiation of naive embryonic stem cells (ESCs) cultured in controlled conditions provides a tractable system to dissect and understand the process of exit from naive pluripotency and entry into lineage specification. Exploitation of this system in recent large-scale RNAi and mutagenesis screens has uncovered multiple new factors and modules that drive or facilitate progression out of the naive state. Notably, these studies show that the transcription factor network that governs the naive state is rapidly dismantled prior to upregulation of lineage specification markers, creating an intermediate state that we term formative pluripotency. Here, we summarize these findings and propose a road map for state transitions in ESC differentiation that reflects the orderly dynamics of epiblast progression in the embryo.

## 1. Introduction

The epiblast is the founder tissue that gives rise to the entire fetus in amniotes. The mouse epiblast segregates as a group of 10–20 cells within the inner cell mass (ICM) of the mature blastocyst around embryonic day (E) 4.0–4.5. Each epiblast cell is considered fully capable of engendering all lineages of the fetus: ectoderm, endoderm, mesoderm and germline. This state of broad developmental plasticity has been called ‘naive pluripotency’ [1]. Epiblast cells isolated at this transitory stage of development can self-renew *ex vivo* and be propagated as cell lines that are called embryonic stem cells (ESCs) [2,3]. Mouse ESCs (mESCs) retain the pluripotent character of the naive epiblast; after extended passaging, clonal cultures can still differentiate into multiple cell types *in vitro*. Furthermore, when introduced into a preimplantation host embryo, they can re-integrate into the developmental programme and contribute to all lineages including the germline to form healthy chimaeric animals.

Emergence of more than 200 specialized cell types in the body from a small number of equivalent cells is a fascinating process and presents a number of fundamental questions to developmental biologists: (1) How is naive pluripotency established? (2) How does pluripotency evolve to enable differentiation? (3) How are lineage decisions made? Here, we focus on the second question, namely, the exit from naive pluripotency and approach to differentiation.

In the mouse embryo, germ layer specification begins in the postimplantation epiblast prior to the onset of gastrulation (E6.5). In the postimplantation period, epiblast cells may go through a series of transitions that progressively channel them to specific fates. Gene expression and immunostaining data demonstrate that the preimplantation and postimplantation epiblast have distinct gene expression profiles. Many genes have also been identified whose mutation disrupts the egg cylinder or germ layer formation. However, from these data alone it is difficult to deduce causative molecular mechanisms that drive transitions in the epiblast population. Elucidation of these mechanisms in the embryo requires the combination of ‘omic’ approaches with technologies for temporally controlled conditional genetic perturbations and real-time reporters of gene expression and signalling pathway activity. These types of studies are limited by poor accessibility of peri- and postimplantation stages *in utero*, sub-optimal development of early postimplantation embryos *ex vivo* and low cell numbers for high-throughput molecular analyses such as proteomics or chromatin immunoprecipitation (ChIP). Alternatively, transitions in the epiblast may be probed using embryo-derived cell lines provided that an experimental setting is established that can reasonably recapitulate *in vivo* development. ESCs provide the foundation for such approaches. In particular, when cultured in defined conditions known as 2i/LIF, ESCs substantially preserve features of the naive preimplantation epiblast. 2i/LIF comprises serum-free medium in which two selective inhibitors (2i) block mitogen-activated protein kinase (MEK) and glycogen synthase kinase 3 (GSK3) activity and the cytokine leukaemia inhibitory factor (LIF) activates the Stat3 pathway [4–6]. ESCs in 2i/LIF can be used as surrogates of the naive epiblast and interrogated experimentally to uncover molecular mechanisms that govern naive pluripotency and orchestrate transitions towards lineage-restricted cell states.

In this review, we first summarize the progression of the naive epiblast towards a lineage-restricted state during embryonic development. We highlight evidence that ESCs cultured in 2i/LIF are authentic *in vitro* counterparts of the naive epiblast and consider postimplantation epiblast-derived stem cells (EpiSCs) and postimplantation epiblast-like cells (EpiLCs) models in comparison with embryonic populations *in vivo*. We then focus on the exit from naive pluripotency and summarize recent large-scale RNAi and insertional mutagenesis screens that have uncovered candidate factors and molecular mechanisms that drive or consolidate the transition into lineage specification. We also expose and challenge three interlinked notions prevalent in current thinking about differentiation of ESCs: (i) that ESCs directly differentiate into germ layers; (ii) that heterogeneous gene expression prepares ESCs for lineage specification; and (iii) that pluripotency factors also act as lineage specifiers. We argue that these propositions are inconsistent with observations of epiblast progression in the embryo and of defined differentiation of ESCs *in vitro*. Finally, we present a roadmap for epiblast development and propose an intermediate phase of formative pluripotency.

## 2. Transition from naive pluripotency to lineage specification in the embryo

**Figure 1.**
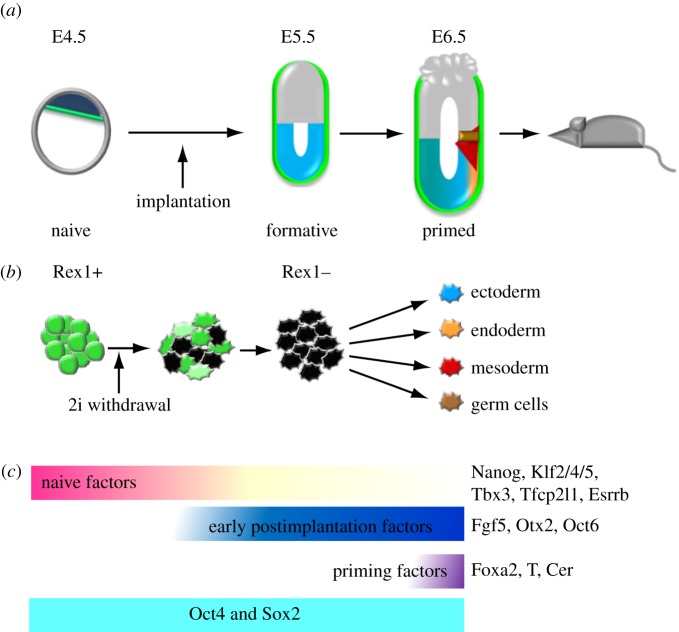
Progression from naive to primed pluripotency. (*a*) Progression of epiblast development in the mouse embryo and corresponding conceptual pluripotent stages. The mature blastocyst comprises three cell lineages: naive epiblast (dark blue), PrE (green) and trophoblast (grey). By E6.5 lineage priming has commenced. Ectoderm, blue; mesoderm, red; definitive endoderm, orange; germline, brown (adapted from Najm *et al.* [8]). (*b*) Progression of naive ESCs to a lineage primed state upon 2i withdrawal. Rex1 is asynchronously downregulated and exit from the naive state is marked with loss of Rex1. These Rex1-negative cells might resemble the early postimplantation epiblast, the intermediate formative stage from which lineage-specified cells emerge. (*c*) Expression periods of naive, early postimplantation and priming factors together with Oct4 and Sox2 during pluripotency transitions.

Between embryonic day 4 and 5, the mouse ICM segregates into two compartments: the naive epiblast and the hypoblast or primitive endoderm (PrE). The epiblast subsequently develops into the embryo proper, whereas PrE gives rise to extraembryonic yolk sac tissues. Naive epiblast cells have several distinctive features. Notably, they can be transferred between embryos and contribute to all lineages in chimaeric mice. They can also self-renew when placed in culture in 2i/LIF, conditions in which Erk/MAPK signalling is completely inhibited. Both of these features are lost upon implantation consistent with a state transition [7]. The naive epiblast expresses general pluripotency factors Oct4, Sox2 and Sall4, but is distinguished from postimplantation epiblast by a suite of transcription factors including Nanog, Klf2/4/5, Tfcp2l1, Tbx3, Esrrb and Rex1 (gene name: *Zfp42*) (figure 1*a*,*c*) [7,9,10]. The latter are called naive markers. Shortly after implantation, the amorphous epiblast undergoes a morphogenetic transformation into a columnar epithelium that in rodents assumes a cup-shape known as the egg cylinder. Molecular landmarks of this transition are suppression of naive markers and upregulation of Fgf5 expression [7,11]. Oct4 and Sox2 continue to be expressed uniformly throughout the epiblast with no significant change in levels until the onset of gastrulation [12]. Importantly, lineage-specific genes such as *T*, *Foxa2* and *Cer* are not initially expressed in the egg cylinder [7] (figure 1*a*,*c*)*.* Subsequently, the postimplantation epiblast starts to become regionalized on day 7 in response to localized expression of secreted factors Nodal, Wnt3 and Bmp4, and their antagonists such as Cer1 and Lefty1 [13]. Signalling pathways and transcription factors downstream of these factors orchestrate formation of lineage-specific gene expression patterns [14], and epiblast cells at the primitive streak stage are considered to be ‘primed’ for lineage commitment [1]. An important conclusion from these observations is that loss of naive pluripotency upon implantation precedes lineage priming. Gene expression analyses indicate that the immediate postimplantation epiblast is devoid of both naive pluripotency factors and lineage-specifying factors [7]. Acquisition of lineage specification occurs over the subsequent 24–48 h implying that the epiblast undergoes further transitions during this time.

## 3. Capture of naive pluripotency *in vitro*

Originally mESCs were derived and cultured in media containing fetal calf serum (FCS) and on feeder layers of mitotically inactivated mouse fibroblasts. Over time, feeders were replaced with LIF and today most commonly used media include LIF and 10–15% FCS, although feeders are still widely used. Importantly, however, ESCs lines that can be efficiently derived and maintained in LIF and serum (LS) are largely from the 129 inbred mouse strain. Moreover, mESC populations in LS without feeders are mosaic [15–19]; individual cells express significantly different levels of naive pluripotency genes and some cells are devoid of naive marker expression. These cultures also express various lineage-specific genes in a heterogeneous manner indicating that a proportion of cells undergo priming and/or differentiation in response to the complex mix of serum components. It has been proposed that ‘metastability’, encompassing heterogeneous expression of lineage-specific genes and pluripotency factors simultaneously across the culture, reflects an inherent fluidity of the pluripotent state and underpins pluripotency by preparing ESCs for lineage commitment [20,21]. However, this mode of fluctuating gene expression has not been persuasively demonstrated *in vivo*, neither in the naive epiblast nor during postimplantation epiblast stages. In particular, germ layer markers are not coincident with naive markers. Moreover, in our experience the variance in gene expression and level of spontaneous differentiation in LS cultures is strongly influenced by serum batch, cell density, feeder cells and genetic background. Therefore, we consider that ESC heterogeneity is a result of sub-optimal culture conditions and does not recapitulate epiblast behaviour *in vivo*. Furthermore, heterogeneity and partial differentiation of starting ESC cultures compromises attempts to chart developmental progression during *in vitro* differentiation.

The advent of 2i culture in 2008 has changed the picture [5]. 2i/LIF enables ESC derivation from all mouse and rat strains tested [22–25] and from single preimplantation epiblast cells with high efficiency [7]. We infer that preimplantation epiblast cells can convert seamlessly into ESCs in 2i/LIF conditions. Moreover, gene expression profiles and DNA hypomethylation states of ESCs in 2i/LIF are similar to preimplantation naive epiblast cells [7,26,27]. These findings suggest that 2i/LIF may provide a signalling environment similar to that experienced by the epiblast in the blastocyst, thereby allowing direct capture and preservation of naive pluripotency *in vitro*. Indeed, naive epiblast cells in the embryo express little or no FGF receptor [7,10,28], rendering these cells unresponsive to the major MEK/ERK stimulus Fgf4 [29]. Nonetheless, it would be informative to examine activity of MEK/ERK signalling directly by immunostaining for active, phosphorylated ERK and by measuring expression of MEK/ERK transcriptional targets.

Although combination of LIF with the 2i inhibitors maximizes the efficiency of ESC derivation and clonogenicity of established ESCs, for most ESC lines any two of these three components is sufficient to maintain the naive state [6,30]. Importantly, ESC populations show substantially uniform expression of key pluripotency factors under each of these conditions, and the heterogeneity and spontaneous differentiation that is characteristic of ESCs cultured without inhibitors or feeder cells in LIF and serum is eliminated [16]. Differentiation of naive ESCs is rapidly initiated when 2i/LIF components are removed, driven by high autocrine expression of Fgf4 [16,30–33]. Efficient progression of naive ESCs into all lineages despite lacking lineage priming or dynamic pluripotency factor expression seems difficult to reconcile with an obligate role for metastability, or dynamic heterogeneity, in pluripotency.

## 4. Tracking exit from naive pluripotency using the Rex1 : GFPd2 reporter

Although naive ESC cultures in 2i/LIF are rather homogeneous, their differentiation is not synchronized. To monitor early phases of differentiation, we adopted *Rex1* (*Zfp42*) as a neutral read-out of naive pluripotency because of its known downregulation after implantation [7,11] and because homozygous deletion of *Rex1* has no phenotypic consequence for ESCs or the mouse embryo [34,35]. We generated a Rex1:GFPd2 knockin reporter ESC line, in which expression of destabilized green fluorescent protein with a half-life of 2 h (GFPd2) is driven by the endogenous *Rex1* promoter [4]. This reporter enables near real-time monitoring and fractionation of early differentiation stages in ESCs by flow cytometry. In monolayer 2i culture with or without addition of LIF, Rex1:GFPd2 shows tight unimodal expression [30–33]. Upon withdrawal of inhibitors, Rex1 : GFPd2 is downregulated in an asynchronous manner resulting in a substantial proportion of Rex1-negative cells in fully defined conditions without exogenous inducers. This occurs within 24 h when beginning with ESCs in 2i. Presence of LIF in the starting culture delays the emergence of Rex1-negative cells by more than 12 h because LIF confers additional stability to the naive state [30]. Loss of Rex1 expression in isolated periimplantation epiblast cells and differentiating ESCs strongly correlates with loss of clonogenicity in 2i/LIF [7,16] (T. Kalkan 2014, unpublished data), indicating that downregulation of Rex1 marks irreversible exit from the naive state. Pluripotency transitions in ESCs based on Rex1 : GFPd2 expression are depicted in figure 1*b*. Expression of Rex1 : GFPd2 has been used as a proxy of the naive state in recent RNAi and mutagenesis screens designed to identify the drivers of entry into differentiation [32,33]. Persistent self-renewal capacity of mutated ESCs that failed to downregulate the reporter in these screens has further corroborated Rex1 expression as a reporter of the naive state [32]. Notably, expression levels of other naive markers such as Nanog, Klf2 and Tfcp2l1 start to decline much earlier than Rex1, approximately 4 h after inhibitor withdrawal [32], yet self-renewal capacity is fully retained as long as Rex1 is maintained (T. Kalkan 2014, unpublished data). Thus, downregulation of key transcription factors such as Nanog, Klf2 or Tfcp2l1 is not sufficient for exit of ESCs from the naive state. This is consistent with previous evidence that a proportion of ESCs that have downregulated Nanog in LS are able to revert to a Nanog-positive state and remain undifferentiated [17,36]. As homozygous deletion of Rex1 is inconsequential for ESC self-renewal and the naive epiblast, we infer that coincidence of Rex1 downregulation with loss of naive state self-renewal is not due a critical function for Rex1 in the naive state. It most probably reflects the cumulative loss of positive transcriptional input from naive pluripotency factors on the Rex1 gene regulatory regions [37]. Additionally, accumulation of a transcriptional repressor might impede Rex1 expression.

Fractionation of differentiating Rex1 : GFPd2 ESCs by flow cytometry might provide an opportunity to isolate emerging Rex1-negative ESCs which are close to the point of exit from the naive state. Characterization of fractionated ESCs at different time points along the differentiation time course might illuminate the order of early molecular transitions in the naive epiblast. In addition, to what extent the early Rex1-negative population resembles the newly implanted or intermediate epiblast is of great interest.

## 5. Generation of postimplantation epiblast *in vitro*: EpiSCs and EpiLCs

The egg cylinder can also give rise to stem cell lines, which are called postimplantation epiblast-derived stem cells (EpiSCs). These cell lines can be derived from a range of postimplantation stages (E5.5 to E8) using basal medium supplemented with activin, Fgf2 with optional addition of KSR (knockout serum replacement) and feeders [38–41]. Since their first derivation in 2007, EpiSCs have been proposed as *in vitro* counterparts of the postimplantation epiblast. EpiSCs express Oct4 and Sox2, but do not express naive pluripotency factors except for Nanog. By contrast, they express the early postimplantation epiblast marker Fgf5, as well as lineage-specific factors, such as T, Foxa2 and Cer. However, these genes are expressed in a highly heterogeneous manner [38,42]. EpiSCs have been shown to retain functional properties of the postimplantation epiblast, in that they can be differentiated to somatic lineages *in vitro* and in teratomas. EpiSCs can also colonize somatic lineages to some extent when injected into postimplantation stage embryos *in vitro* [38,43], though not when introduced into preimplantation embryos. These differentiation assays have not been performed using single cells, and recent evidence suggests that EpiSCs comprise a mixed population of lineage progenitors cells along with pluripotent precursors [44].

EpiSCs can also be generated from ESCs by differentiation in the continuous presence of activin and Fgf2 [45]. However, stable EpiSC cultures are obtained only after passaging and the process is accompanied by heterogeneous differentiation and cell death. This suggests that ESCs do not directly convert into EpiSCs, but that a divergence from the normal differentiation path is required. In line with this idea, a recent study which compared several independently derived EpiSC lines to different postimplantation epiblast stages revealed that the transcriptome of EpiSC lines was variable but resembled most closely late gastrulation stage epiblast, regardless of the original embryo stage from which the cell lines were derived [38]. Induction of the EpiSC state by culture components is further indicated by derivation of EpiSCs from blastocyst explants [46]. When injected into postimplantation stage embryos, EpiSCs most efficiently integrate in the anterior primitive streak. Overall, their heterogeneity, late epiblast characteristics, and protracted derivation from ESCs reduce the utility of EpiSCs for studying pluripotent transitions.

However, transitional cells with similarities in gene expression profile to early postimplantation epiblast (E5.5) have been identified during differentiation of ESCs. These postimplantation epiblast-like cells (EpiLCs) are transient and form from naive ESCs at around 48 h after withdrawal of 2i/LIF and addition of EpiSC medium containing KSR [8,47]. EpiLCs do not exhibit naive pluripotency gene expression, but express early postimplantation epiblast markers such as Fgf5, Otx2 and Oct6 along with Oct4 and Sox2. Transcriptome profiling places them close to the early postimplantation epiblast and distinguishes them from EpiSCs which express later lineage markers. Consistent with this identity, and unlike EpiSCs, EpiLCs can be differentiated efficiently to primordial germ cells and give rise to functional gametes, a property of the pre-gastrulation intermediate epiblast [47–49].

## 6. The molecular route to exit from the naive embryonic stem cell state

The naive state of ESCs and the preimplantation epiblast is characterized by co-expression of a set of transcription factors called the ‘naive pluripotency factors' (figure 1*c*). These naive factors together with Oct4 and Sox2 constitute the transcriptional control circuitry of the naive state [7,16,30]. The network is maintained cooperatively through cross-regulation, generating a self-reinforcing regulatory circuit when insulated from MEK/ERK and GSK3 activity. LIF confers additional robustness to the network through upregulation of Tfcp2l1 and Klf4 [19,50,51]. When ESCs maintained in 2i without LIF are withdrawn from inhibitors, a decline in transcript levels of naive factors is evident from as early as 4 h [32]. Naive pluripotency factor expression is largely eliminated within 24 h and the network is eliminated entirely by 48 h, accompanied by loss of self-renewal ability in 2i/LIF [30–32]. Coincident with dismantling of the naive pluripotency network, characteristic markers of the postimplantation epiblast such as Fgf5, Oct6 and Otx2 are induced and de novo methyltransferases Dnmt3a1 and Dnmt3b are upregulated (T. Kalkan 2014, unpublished data). Overall, these gene expression changes appear very similar to those observed during differentiation of 2i/LIF-cultured ESCs by withdrawal of 2i/LIF with or without addition of exogenous factors such as activin or Fgf2, suggesting that the major driver of this initial transition is activity of MEK/ERK and GSK3 [8,48]; (T. Kalkan 2014, unpublished data).

Monolayer differentiation of naive ESCs following 2i withdrawal has been exploited in large-scale mutagenesis and RNAi screens to find molecular drivers and facilitators of the exit from the naive state. Hits were identified based on persistence of Rex1 : GFPd2 expression, retention of self-renewal ability in 2i/LIF, or both. The design of these screens is summarized in table 1. Overall, 600 protein-coding genes have been identified in three screens [31–33]. Interestingly, the vast majority of these are expressed in the naive state, with only 69 being transcriptionally upregulated upon 2i withdrawal (T. Kalkan 2014, unpublished data). This finding indicates that many regulators might be idle in the naive state owing to the absence of active MEK/ERK and GSK3 signalling, or their functions might be counteracted directly by naive pluripotency factors. An implication from these data is that ESCs, and by analogy naive epiblast cells, are intrinsically poised for developmental progression. This may underlie the rapid transitions during periimplantation development in rodent embryos.

**Table 1.** Summary of large-scale exit from naive pluripotency screens.

reference	loss-of-function method	coverage	no. of high confidence hits	differentiation method	criteria for selection of positive hits
Betschinger *et al.* [31]	siRNA	9900 genes	28	monolayer differentiation in N2B27 for 96 h	proliferation in 2i/LIF and retained Oct4 expression
Yang *et al.* [33]	siRNA	genome-wide	272	monolayer differentiation in N2B27 for 28 h/96 h	retained Rex1:GFPd2/Oct4:GFP expression
Leeb *et al.* [32]	haploid insertional mutagenesis	genome-wide	113	two rounds of monolayer differentiation in N2B27 for 7–10 days	proliferation in 2i/LIF and retained Rex1:GFPd2 expression

Fgf4/MEK/ERK has been identified as the major signalling cascade that initiates ESC differentiation. Genetic deletion of the secreted ligand Fgf4 or the MEK target ERK2 mimics MEK inhibition and impedes ESC differentiation along multiple lineages [52,53]. In the embryo, lack of Grb2, which couples the Fgf receptor to the MEK/ERK pathway, or chemical inhibition of MEK converts the entire ICM into Nanog-positive epiblast at the expense of the hypoblast, indicating that naive pluripotency *in vivo* arises in the absence of MEK/ERK signalling [54,55]. Consistent with these observations, multiple core components of the Fgf4/MEK/ERK signalling cascade including Fgfr2, Raf1, K-Ras, N-Ras, MEK2 and ERK1/2 were recovered in the exit from naive pluripotency screens; however, downstream targets of ERK1/2 that are critical for ESC differentiation still await identification. ERK1/2 have more than 200 known substrates with diverse functions [56,57], hence multiple proteins identified in the differentiation screens are expected to be direct phosphorylation targets of ERK1/2.

**Figure 2.**
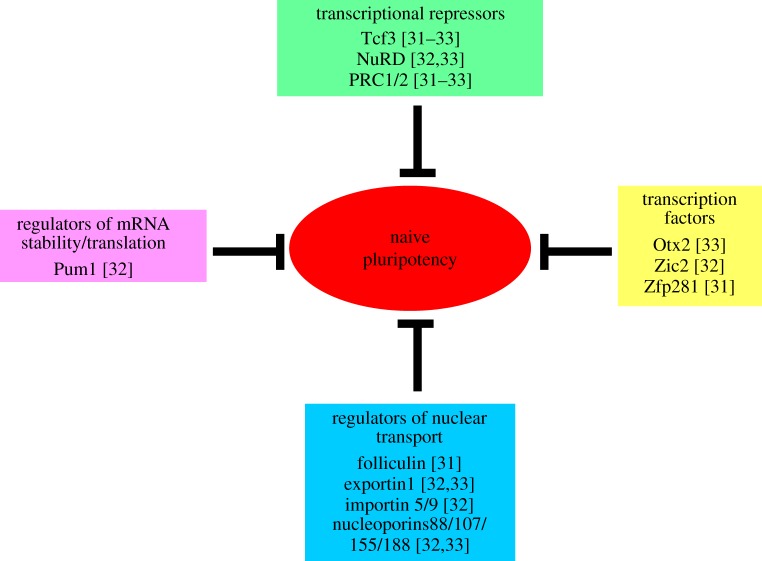
Negative regulators of naive pluripotency. In the presence of active MEK/ERK and GSK3, the naive pluripotency factors are subject to coordinated attack at different levels: transcriptional repression, mRNA stability and translation, nuclear/cytoplasmic localization. Induced/activated transcription factors Otx2, Zic2 and Zfp281 might cooperate with these mechanisms to further suppress the levels of naive factors. They also activate new genes that mediate further developmental progression.

Functional examination of validated hits and candidates emerging from the screens indicates that timely dismantling of the naive pluripotency network and acquisition of a postimplantation epiblast-like gene expression profile is ensured by a combination of mechanisms acting at multiple levels (figure 2). Below we discuss some of the major mechanisms regulating the levels of naive pluripotency factors, including transcriptional regulation, nuclear transport and mRNA stability. We also consider an emerging mechanism of transcription factor repurposing to establish new gene expression programmes during developmental transitions.

## 7. Repression of naive pluripotency factor transcription

Tcf3 (gene name: *Tcf7l1*) is a transcriptional repressor implicated in Wnt signalling and has been shown to be the main downstream effector of GSK3 signalling in ESC differentiation. Knockout or mutation of Tcf3 renders ESCs largely resistant to differentiation [4,58,59]. The pivotal role of Tcf3 in exit from the naive state has been reinforced by identification of Tcf3 as the top hit in all three screens [31–33]. Upon withdrawal of Chiron, derepressed GSK3 phosphorylates β-catenin and causes its proteosomal degradation. Mechanistic studies showed that degradation of intracellular β-catenin frees Tcf3 to exert its repressor function [60]. Naive pluripotency factors Klf2, Nanog and Esrrb are all directly repressed by Tcf3 [4,59–61]; however, among these repression of Esrrb appears to be the most critical for exit from naive pluripotency upon Chiron withdrawal [61]. Interestingly, ChIP studies showed that Tcf3 is largely localized to the same genomic loci as Oct4 and Nanog in serum-cultured ESCs [62]. However, it is hard to evaluate whether the three factors co-occupy these loci simultaneously, as serum-cultured ESCs comprise cells in different states. Nevertheless, this finding raises the possibility that Tcf3 might be deployed to key naive pluripotency genes together with Oct4, which might underlie its potent repressive action upon 2i withdrawal. The recently described interaction of Tcf3 with Oct4 in naive cells strengthens this possibility [8].

Another transcriptional repressor recovered in the recent screens is the nucleosome remodelling and histone deacetylation (NuRD) corepressor complex. A requirement for NuRD in early differentiation phase of ESCs and the naive epiblast is well established [63,64]. Abolishing NuRD function through genetic deletion of its scaffold protein Mbd3 was shown to cause upregulation of naive pluripotency transcripts in ESCs, with most pronounced effects on Tbx3 and Klf4 [65]. Consequently, *Mbd3*-null ESCs exhibit less spontaneous differentiation in LS and can self-renew in the absence of LIF. When injected into blastocysts, these cells do not contribute to postimplantation development. *Mbd3*-null embryos fail shortly after implantation consistent with a requirement for NuRD during exit from the naive state. In line with these previous findings, several components of the NuRD complex including Mbd3, Gatad2, Mta1 and Rbbp4 were identified as candidate factors that drive exit from the naive state in ESCs, placing NuRD as a key transcriptional repressor required for termination of the naive pluripotency gene expression programme (figure 2). NuRD was shown to regulate gene expression by deacetylating lysine 27 of histone 3 (H3K27) and thereby facilitating polycomb repressive complex 2 (PRCR2)-mediated trimethylation of H3K27 and associated gene repression [66].

Zfp281, a transcription factor that has been identified in an siRNA screen [31], was proposed to recruit the NuRD complex to the *Nanog* promoter through direct interaction with Nanog and NuRD components [67–70]. Moreover, *Zfp281*-null ESCs were shown to exhibit increased Nanog and Rex1 expressions and could not be differentiated into embryoid bodies. *Zfp281*-null embryos are reported to die around E8 although the phenotype is not detailed [71]. Zfp281 thus appears to be another significant player in exit from naive pluripotency, although its precise mode of action merits further investigation.

Finally, epigenetic silencers, the polycomb repressive complexes (PRC1 and PRC2), are implicated in exit from the naive state. PRC1 components Phc1 and Ring1B, and PRC2 components Ezh2, Suz12, Mtf2 (Pcl2) and the PRC2-associated protein Jarid2 were recovered in the screens (figure 2). PRC1 and PRC2 secure transcriptional repression by introducing chromatin compaction through mono-ubiquitylation of lysine 119 of histone 2A (H2AK119ub) and trimethylation of lysine 27 of histone 3 (H3K27me3), respectively [72]. Single and double knockout of these complexes demonstrated that they function semi-redundantly in ESCs [73]. While knockout of either PRC1 or PRC2 by genetic deletions of their respective core catalytic enzymes, Ring1B and Ezh2, only mildly affected self-renewal or differentiation of ESCs, *Ring1B*/*Ezh2* double null ESCs could not execute differentiation. Notably, these cells were able to self-renew, suggesting that the critical role of PRC complexes is during early differentiation. This proposition is now further strengthened by failure of cells that are compromised in PRC activity to exit the naive state efficiently in the genetic screens. Consistent with this, the number of genes marked with H3K27me3 and the intensity of this repressive histone mark is significantly reduced in naive ESCs compared with LS-cultured ESCs without an apparent difference in expression of PRC2 components [16], suggesting that H3K27me3 marks are associated with differentiating cells in LIF/serum and PRC2 activity might be enhanced downstream of active MEK/ERK and GSK3 signalling to secure timely exit from the naive state.

## 8. Control of naive pluripotency factor transcription via nuclear/cytoplasmic transport

In addition to direct repression by Tcf3, transcription of the potent naive pluripotency factor Esrrb is further reduced by the activity of the folliculin/Fnip complex. The unexpected role of folliculin/Fnip emerged from an siRNA screen [31]. This complex regulates subcellular localization of the basic helix-loop-helix transcription factor Tfe3, which was shown to bind to *Esrrb* cis-regulatory regions together with Oct4 and Nanog and contribute to positive regulation of *Esrrb* transcription. In naive ESCs, Tfe3 protein is distributed in both the nucleus and cytoplasm, but upon 2i withdrawal, nuclear Tfe3 is sequestered in the cytoplasm in a manner dependent on folliculin/Fnip. Thus, access of Tfe3 to its transcriptional targets, notably *Esrrb*, is prevented. Importantly, Tfe3 protein is excluded from the nuclei of epiblast cells after implantation indicating that the same mechanism is likely to be operation *in vivo*. It is not known how the activity of folliculin/Fnip complex is regulated downstream of MEK/ERK and GSK3, although mTOR clearly plays a role [31]. Nor is it clear how folliculin/Fnip causes nuclear exclusion of Tfe3. However, recovery of factors with roles in nuclear/cytoplasmic transport in the loss-of-function screens suggests that selective nuclear import and export of transcription factors might be a more general mechanism that regulates exit from the naive state (figure 2). Moreover, it has been shown that 2i/LIF withdrawal induces auxetic properties in the nuclei of naive ESCs, meaning the nuclei increase in volume when stretched and stiffen when compressed under physical forces [74]. This structural change was found to be associated with chromatin decondensation; however, it might also impact on the rates of simple diffusion and selective nuclear/cytoplasmic transport of signalling molecules to affect exit from the naive state.

## 9. Stability of naive pluripotency factor transcripts

Reduction in the levels of naive pluripotency factor mRNAs is remarkably rapid, declining by 70% only 4 h after 2i withdrawal. This fast response suggests that mechanisms other than transcriptional control at the promoter level may play a role. Consistent with this idea, Pum1, which is an RNA-binding protein that inhibits translation and promotes degradation of target mRNAs [75,76], was identified in an insertional mutagenesis screen [32]. Pum1 knockdown in ESCs impaired exit from the naive state and delayed downregulation of transcripts for Tfcp2l1, Klf2, Tbx3, Esrrb, Sox2 and Nanog [32]. Importantly, Pum1 was found to physically interact with mRNAs for naive factors. Thus, Pum1 most probably directly promotes their degradation upon 2i withdrawal and potentially also inhibits translation. Furthermore, Pum1 activity appears to be constitutive because in 2i conditions it is bound to its target mRNAs and knockdown leads to mild upregulation. Hence, constitutive function of Pum1 might constrain self-renewal in addition to ensuring rapid elimination of translation and clearance of transcripts when transcription is reduced. Whether Pum1 activity is further enhanced in response to MEK/ERK and GSK3 is currently not known. In either case, it seems that Pum1-mediated mRNA degradation is a further mechanism that ensures rapid dissolution of the naive pluripotency network. It will be interesting to see if this mechanism is reused in other cell fate transitions.

## 10. Initiation of a new transcription programme

As the naive pluripotency network is dissolved within the 48 h following 2i/LIF withdrawal, hundreds of new genes are induced and the cells acquire a transcription profile resembling early postimplantation epiblast [8,33,47]. An unresolved question is how cells initiate a new transcription programme. Is dismantling of the naive pluripotency network sufficient for activation of the differentiation programme if some of the naive factors directly repress differentiation-associated genes? Or does this process require recruitment of new transcription factor complexes to gene regulatory regions that are activated during the transition? Recent studies shed some light onto these questions by showing that Oct4 and transcription factors induced early upon 2i withdrawal, such as Otx2, cooperatively induce new gene expression during the transition [8,77]. Both Oct4 and Otx2 were recovered in an siRNA screen as candidate factors to drive exit from the naive state [33]. Oct4 was previously implicated in early differentiation because cells with reduced levels of Oct4 failed to differentiate efficiently and showed enhanced self-renewal [78,79]. Otx2 was also reported to be required for early differentiation of ESCs and conversion to EpiSCs as well as for postimplantation development [80,81]. Building on these observations, recently two independent studies uncovered a mechanistic link between Oct4 and Otx2 which delineates one of the routes ESCs use to initiate a new gene expression profile upon MEK/ERK and GSK3 activation [8,77]. The principal finding of these studies is that Otx2 is rapidly induced after 2i withdrawal and recruits Oct4 to enhancers that are associated with genes induced during differentiation. Moreover, both studies implicate Otx2 in the activation of these enhancers. Importantly, in the EpiLC differentiation protocol, Oct4 was found to interact with different sets of transcription factors and chromatin modifiers in the naive state and in EpiLCs, while other interaction partners, such as Sox2, were common in both states [8]. In the naive state, Oct4 pull-down complexes contained Tcf3, Esrrb and Klf5, whereas in EpiLCs, Oct4 was found to co-purify with Otx2, Zic2/3, Oct6, Zfp281 and Zscan10 [8]. Thus, Oct4 is likely to function in different transcription factor complexes that are specific to the naive and EpiLC states. Notably, two Oct4-interacting proteins specific to EpiLCs, Zic2 and Zfp281, were also identified in the exit from pluripotency screens [31,32], suggesting that Oct4 might engage with these proteins in a fashion similar to its partnership with Otx2 to affect new transcription. Oct4 partner switch may be dictated by the relative levels of potential Oct4 partners in a cell. As a way of example, in the naive state Esrrb is highly expressed and Otx2 is low; therefore, by simple mass action Oct4 may be more likely to form a protein complex with Esrrb. Upon 2i withdrawal, Esrrb is downregulated and Otx2 is induced which may favour formation of Oct4–Otx2 complexes. Such an Oct4 partner switch can result in differential enhancer selection, as has been demonstrated by artificially modulating the levels of two Sox family transcription factors, Sox2 and Sox17, both of which can interact with Oct4 [82]. Differential partner interaction may account for the Oct4 overexpression phenotype. Upregulation of Oct4 was reported to cause mesoendodermal differentiation in LS-cultured ESCs, and to accelerate neural differentiation in serum-free LIF-deficient medium [83,84]. These observations together with persistence of Oct4 and Sox2 during early differentiation subsequently led to the proposal that these transcription factors act as lineage specifiers [85]. Furthermore, differentiation phenotypes associated with Sox2 and other pluripotency factors in mouse and human ESCs led to a hypothesis that all pluripotency factors are lineage specifiers and pluripotency is a precarious balance between opposing lineages [86]. However, some of the observations behind this hypothesis can be explained by promiscuous partner selection by Oct4. In differentiation permissive medium such as LS, in which differentiation-associated partners of Oct4 such as Otx2 and Sox17 are expressed, overexpression of Oct4 might increase interactions with these factors and promote differentiation. However, as such partners are not expressed in the naive state, moderate overexpression of Oct4 in 2i/LIF may be compatible with self-renewal. This remains to be tested. Interestingly, Sox2 was also identified as a factor required for exit from the naive state [31], raising the possibility that like Oct4, Sox2 might switch partners to promote differentiation. Both Oct4 and Sox2 continue to be expressed at the postimplantation stages and here they are likely to play roles in lineage specification. Whether these are really opposing remains to be experimentally validated.

## 11. Discussion and conclusion

More than 600 proteins have been implicated in exit from the naive state. Here, we have discussed only a handful, primarily, of those whose function has been independently validated. However, this limited set is sufficient to reveal the variety of the mechanisms that control early state transitions in ESCs and complexity of the regulation. Investigation of further candidates will expand our understanding in this area and may facilitate efforts in deriving naive ESCs from non-rodent species, including humans. The fact that these actors are revealed upon 2i withdrawal but are mostly present in the naive state suggests that ESCs cultured in LIF/serum are likely to be continuously challenged owing to active MEK/ERK and GSK3 signalling induced by undefined serum components in addition to autocrine Fgf4. In this light, the widely commented upon heterogeneity of ESCs in LS appears to be a response to an incoherent signalling context, in which the naive pluripotency circuitry is constantly confronted with differentiation stimuli. We contend that the defined 2i/LIF culture system for ESCs provides a more appropriate and accurate reflection of naive epiblast in the embryo. ESC differentiation after 2i/LIF withdrawal mimics orderly transitions of the naive epiblast upon implantation and provides a relevant simulation of the early lineage specification process. Elucidation of regulatory mechanisms that redeploy Oct4 during the transition confirms that the central element of pluripotency drives developmental progression, and hence reliable capture of the naive state requires LIF reinforced by MEK/ERK and GSK3 inhibition [7,30].

Presence of naive pluripotency factors appears to create and enforce an unrestricted state in the pre-implantation epiblast and ESCs. Clearance of these factors subsequent to implantation *in utero* or withdrawal of 2i/LIF *in vitro* paves the way for differentiation. Upon downregulation of naive pluripotency factors, pluripotent cells become receptive to differentiation stimuli and exhibit competence to form all somatic lineages and the germline. We propose that this formative state devoid of both naive factors and lineage specifiers is a necessary predecessor to orderly lineage specification. Figure 1 depicts this conceptual framework in which the direct successor of naive pluripotency is a state of ‘formative pluripotency’ enabled for lineage priming. EpiLCs and Rex1-negative cells generated by monolayer differentiation without exogenous factors appear related to this intermediate stage. Although information is currently limited on the homogeneity of these populations, refined culture conditions and fractionation via reporter systems might yield populations representative of formative epiblast. We speculate that the absence of inhibition by naive factors and of bias introduced by lineage specifiers may render epiblast cells optimally receptive for homogeneous and efficient differentiation into any embryonic lineage. Accordingly, it would be of great interest to capture and propagate cells in such a state of formative pluripotency.
